# The neurobiology of love and addiction: Central nervous system signaling and energy metabolism

**DOI:** 10.3758/s13415-025-01333-w

**Published:** 2025-08-04

**Authors:** Tobias Esch, George B. Stefano

**Affiliations:** 1 Institute for Integrative Health Care and Health Promotion (IGVF), Faculty of Health/School of Medicine, Witten/Herdecke University, Witten, Germany; 2 Department of Psychiatry, First Faculty of Medicine, Charles University and General University Hospital in Prague, Ke Karlovu 11, 120 00 Prague, Czech Republic

**Keywords:** Reward pathway, Dopamine, Drug addiction, Nitric oxide, Relaxation response, Bioenergetics, Broken heart, Stress, Brain, Romantic love

## Abstract

Despite our ongoing fascination with love’s pleasures and pain, psychologists and neurobiologists have only recently begun to explore the neurobiological connections shared by feelings of romantic love and the experience of drug addiction. Functional imaging studies have revealed that feelings resulting from romantic love and those resulting from active drug use both activate the central reward system, which is a series of forebrain and midbrain structures that transmit signals primarily via dopamine release. Similarly, the relaxation response, which is a series of behaviors designed to alleviate stress-related physiologic sequelae, may also be helpful as an adjunct therapy for drug withdrawal. The benefits of the relaxation response and related mind-body practices may stem directly from its impact on mitochondria, organelles that are central to balanced energy production. Nitric oxide (NO) is a central neurotransmitter and also a key regulatory molecule that modulates mitochondrial respiration and oxygen utilization. Thus, we propose that observed behaviorally mediated changes that emerge from engaging the relaxation response may be the result of NO-mediated improvements in mitochondrial bioenergetics. Future research might focus on elucidating the important links between cellular bioenergetics, the relaxation response, and the central reward system and might explore NO modulation as a potentially effective target for drug development.

*Love is like a narcotic. At first it brings the euphoria of complete surrender. The next day, you want more*.

– Paulo Coelho (1994)

## Introduction

We are all familiar with the profound emotional impact of romantic love. Apart from our own experiences, true love remains among the most frequent themes explored in drama, fiction, music, and poetry since time immemorial. Interestingly, and despite our ongoing fascination with love’s pleasures and pain, psychologists and neurobiologists have only recently begun to explore the physiological connections between romantic love and the experience of drug addiction. In this narrative review, we will discuss our current understanding of this field and suggest some directions for future research.

## Romantic love and addiction

From a psychologist’s perspective, romantic love is a response involving intimacy and passion in which the loved party is often idealized (American Psychological Association, 2018a, 2018b). When requited, romantic love features feelings of mutual appreciation, desire, and excitement, together with a wish for physical proximity. Anthropologist H. E. Fisher and colleagues (2016) discussed romantic love as a “natural addiction” and an evolutionary adaptation that developed together with mechanisms that facilitate long-term bonding and effective child-rearing. By contrast, neurobiologists might define romantic love as a motivational state that involves the neurochemical activation of pleasure centers in the central nervous system (CNS) (Esch & Stefano, 2005a, 2005b; Seshadri, 2016).

At first glance, romantic love and drug addiction (also known as substance use disorder) appear to be completely unrelated phenomena. The psychological literature defines addiction as a state of mental and/or physical dependence on drugs or other substances (e.g., alcohol, tobacco) and/or behaviors (e.g., gambling) (American Psychological Association, 2018a, 2018b). By contrast, the clinical/neurobiological definition focuses on addiction as a chronic relapsing disorder involving complex interactions among brain circuits and the emergence of a negative emotional state (American Society of Addiction Medicine, 2019; Koob & Volkow, 2016).

While these definitions of romantic love and addiction are both formal and distinct, recent studies focused on dissecting brain pathways and neurochemical activation patterns tell another story. Interestingly, in their 1975 book entitled “Love and Addiction,” authors Stanton Peele and Archie Brodsky (1975) were among the first to highlight the links between these two phenomena and consider the possibility of developing novel rehabilitation and treatment strategies based on these observations. While this possibility was discussed phenomenologically in earlier literature, the results of recent mechanistic/imaging studies revealed that both romantic love and addictive substances can activate what is known as the central “reward pathway” (Lewis et al., 2021). The components and activities of the reward pathway and its responses to romantic love and substance use are discussed in the following sections.

## The reward pathway

The reward pathway (also known as the mesolimbic-mesofrontal dopaminergic system) is a complex series of forebrain and midbrain structures that signal to one another to reinforce pleasurable activities. Pleasurable stimuli activate this circuit by triggering the ventral tegmental area (VTA) to generate dopaminergic signals. These signals are received and processed by the nucleus accumbens (NAc) with additional roles played by the prefrontal cortex, the hippocampus, the striatum, and the amygdala (National Institute on Drug Abuse, 2007; Lewis et al., 2021; Esch & Stefano, 2010).

The central reward system is activated in individuals who are experiencing emotions associated with romantic love or engaged in drug use. In the mammalian brain, opiate drugs activate the central reward system by binding to endogenous receptors. While these receptors exist throughout the CNS (Dhaliwal & Gupta, 2023), specific Mu-type opiate receptors are found at high density in the VTA, NAc, and prefrontal cortex. Morphine binds to these receptors and promotes dopaminergic signaling (Valentino & Volkow, 2018; Dhaliwal & Gupta, 2023). Moreover, recent positron emission tomography (PET) and functional magnetic resonance imaging (fMRI) studies have shown that drug use activates central reward circuits, as well as brain regions involved in motivation, memory, and cognitive control (Volkow et al., 2003; Murnane et al., 2023).

**Fig. 1:**
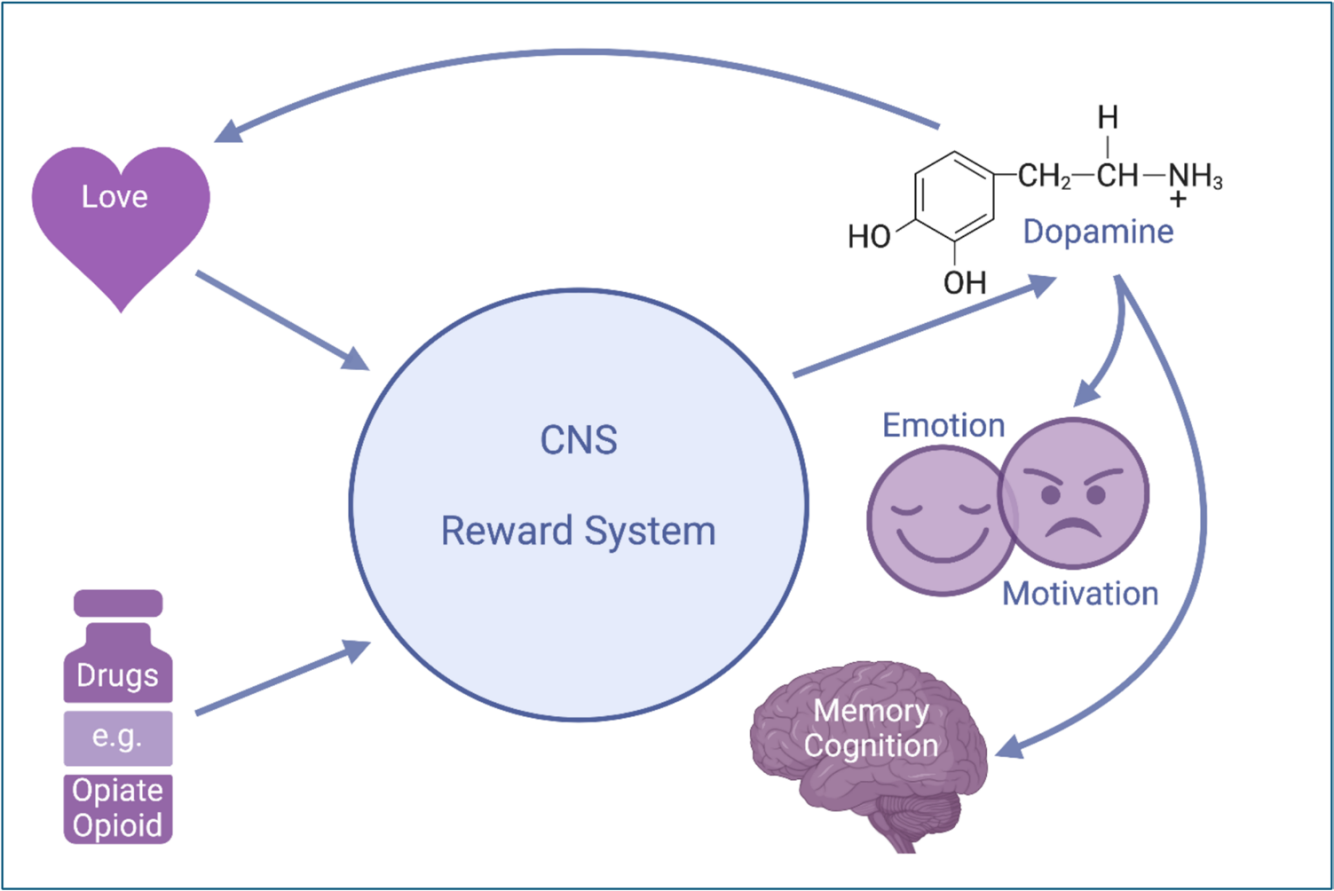
CNS reward system involvement in love, drug administration – motivation, memory regulation. Explanations see text; CNS = central nervous system

Interestingly, functional imaging studies performed on patients experiencing romantic love revealed similar findings (reviewed in Zeki, 2007; Fisher et al., 2005). For example, Aron et al. (2005) performed fMRI studies on both males and females reported to be intensely “in love” and found that exposure to object-specific stimuli led to the activation of dopamine-rich areas associated with mammalian reward and motivation, including the right VTA. Similarly, Song et al. (2015) performed resting state fMRI on a series of volunteers and found increased functional connectivity within the reward, motivation, and emotional regulation networks (including the amygdala, and NAc) among those identified as currently “in love” (Fig. 1). These findings are confirmed by recent studies (Rinne et al., 2024). Similarly intriguing, Burkett et al. (2011) reported that specific blockade of opiate Mu-receptors in the mammalian brain (females of the monogamous prairie vole, *Microtus ochrogaster*) resulted in diminished partner preference.

## Neurotransmitters within the reward pathway

### Dopamine

Dopamine is the primary neurotransmitter involved in reward and motivation, romantic love, pleasure derived from social approval, and responses to substance use. Both D1 and D2-type dopamine receptors have been identified in the NAc, a key region that responds to dopaminergic signaling from the VTA (Lewis et al., 2021). Although dopamine was originally discovered in the 1950 s and characterized using more traditional biochemical methods (Iversen & Iversen, 2007; Carlsson, 2000), PET has been used to explore the contributions of dopamine in the reinforcing effects of drugs, the long-term brain changes in drug-addicted subjects, and factors associated with vulnerability to addiction (Volkow et al., 2007; Tomkins & Sellers, 2001).

Dopamine plays a key intermediary role in a broader biochemical system, including the biosynthesis of endogenous morphine and its interactions with nitric oxide (NO) and nitric oxide synthase (NOS); both of these factors influence mitochondrial function and adenosine triphosphate (ATP) production. Endogenous morphine modulates mitochondrial respiration and redox homeostasis in part via its interactions with NO signaling (Stefano et al., 2012; Stefano et al., 2015). Dopamine, a catecholamine precursor to endogenous morphine in certain cell types, may thus indirectly influence mitochondrial bioenergetics (Zhu et al., 2005). The implication is that dopamine withdrawal (such as that occurring in response to stress or addiction) could disrupt this network, diminishing the efficiency of ATP synthesis and increasing oxidative stress.

### Oxytocin and vasopressin

Oxytocin and vasopressin play critical roles in social bonding and attachment responses. Oxytocin is released during loving interactions, social connections (e.g., feeling appreciated), and even during certain reward-related activities (e.g., trust-building tasks) (Rigney et al., 2022; Carter, 2017; Insel, 2010). Oxytocin receptors are also concentrated primarily in the VTA and NAc, where they modulate dopamine release (Peris et al., 2016). Vasopressin V1a receptors also modulate dopamine signaling in the brain, notably in the ventral pallidum (Carter, 2017; Insel, 2010). While these molecules enhance the emotional salience of rewarding and bonding experiences, they have also been implicated in drug-induced reward responses (Wronikowska-Denysiuk et al., 2023).

### Gamma-aminobutyric acid

Gamma-aminobutyric acid (GABA) is a neurotransmitter synthesized primarily in specific GABAergic neurons that are widely distributed throughout the CNS. Recent technical advances, including the availability of proton magnetic resonance spectroscopy, have led to an improved understanding of the role of this neurotransmitter in health and disease (Shyu et al., 2022). Among its actions in the CNS, GABA modulates the central reward system primarily via its interactions with dopaminergic neurons (Volkow et al., 2019).

### Other agents

Other endogenous signaling molecules that interact with the central reward system with implications for drug addiction include enkephalins and beta-endorphin (reviewed in Rysztak & Jutkiewicz, 2022; Roth-Deri et al., 2008; Le Merrer et al., 2009; Zalewska-Kaszubska & Czarnecka, 2005). The central role of NO as both a neurotransmitter and modulator of bioenergetic balance is discussed below.

## The relaxation response

First described by Herbert Benson & colleagues (1975), the relaxation response is a physical state of deep rest that can alter one’s emotional and physical responses to stress. While long understood as a means to alleviate stress-related physiologic responses (Beary et al., 1974), more recent studies suggest that invoking the relaxation response may be helpful as an adjunct therapy for drug withdrawal (Klajner et al., 1984; Lotfinia et al., 2024) and may even have a positive impact on those suffering from similar symptoms that have developed after a love relationship has ended. In the following sections, we will explore the physiology of the relaxation response with this in mind.

The rationale for the use of relaxation techniques (e.g., slow, diaphragmatic breathing and meditative states) rests on the assumption that they can restore balance in autonomic tone, reduce systemic stress responses, and enhance mitochondrial efficiency independent of direct dopamine replacement. These effects are mediated through reductions in sympathetic activity, normalization of cortisol and inflammatory markers, and upregulation of mitochondrial biogenesis and oxidative phosphorylation (Brown & Gerbarg, 2005; Gautam et al., 2021a; 2021b). Relaxation may also activate vagal pathways that modulate NO production and mitochondrial function, potentially compensating for the metabolic disruptions associated with dopamine withdrawal (Tracey, 2002; Stefano et al., 2012). Thus, while relaxation therapy may not lead to direct restoration of dopamine levels, it can still improve mitochondrial ATP production and reduce stress-related metabolic dysregulation through parallel pathways, thereby supporting its use even in cases of dopaminergic deficiency.

## The impact of the relaxation response on neurotransmitters and metabolic bioenergetics

The health benefits associated with the relaxation response stem from reestablishing a balance between the sympathetic and parasympathetic branches of the autonomic nervous system (Esch & Stefano, 2010). In addition, emerging evidence links relaxation response to enhanced mitochondrial bioenergetics, improved insulin secretion, reduced inflammation, and diminished activation of stress-related pathways (Karrasch et al., 2023; Bhasin et al,. 2013; Picard et al., 2018). However, while these associations suggest adaptive physiological changes, they fail to define a unified mechanism underlying the relaxation response's clinical efficacy. We propose that relaxation response and similar mind-body practices promote systemic metabolic advantages by synchronizing the functions of peripheral organ systems and the CNS to optimize ATP production and energy balance (Stefano et al., 2019). In this regard, it is important to mention that dopamine, the prototype catecholamine, plays a crucial role as a chemical intermediate in endogenous morphine biosynthesis in animals (Stefano et al., 2015). Endogenous morphinergic signaling, alongside NO-coupled systems, has evolved as a modulator of energy metabolism and mitochondrial respiration (Stefano et al., 2015). This concept aligns with the concept of mitochondrial “enslavement” during eukaryotic evolution, a step that was potentially mediated by endogenous morphine (Stefano & Kream, 2017; Bressan & Kramer, 2021).

We hypothesize that the relaxation response and similar mind-body practices may promote systemic advantages by synchronizing the activities of various organs and organ systems (e.g., the respiratory, cardiovascular, and musculoskeletal systems, among others) with the central nervous system (CNS) as a means to optimize ATP production and overall energy balance. Although this idea remains a hypothesis at this time, emerging research supports its biological plausibility. For example, Bhasin & colleagues (2018) reported that practices eliciting the relaxation response resulted in a significant increase in the expression of genes associated with energy metabolism and mitochondrial function, notably mitochondrial ATP synthase. These results suggest that mind-body interventions can directly enhance cellular energy production and potentially its utilization (Dusek et al., 2006; Stefano et al., 2001). Conceptually, the relaxation response may serve as a physiological counterbalance to the stress-induced"fight-or-flight"state by shifting toward parasympathetic dominance—a state characterized by reductions in blood pressure, heart rate, and overall oxygen consumption. Collectively, these responses are indicators of a coordinated physiological alignment that fosters energy efficiency and restorative function across multiple systems (Furlan et al., 2023; Priest & Tontonoz, 2019; Ye & Medzhitov, 2019).

Nitric oxide plays numerous physiologic roles, largely depending on precisely when and how it is produced (reviewed in Stefano et al., 2000). Under homeostatic conditions, small amounts of NO are synthesized continuously by both endothelial and neuronal NOS (eNOS and nNOS, respectively). Constitutive NO synthesis regulates blood flow, supports communication between nerve cells, and protects cells from oxidative damage (Epstein et al., 1993). By contrast, inducible NOS (iNOS) is activated during infections or inflammation. This enzyme, which produces much larger amounts of NO, assists with pathogen elimination. However, if overproduced or if its synthesis is sustained for long periods, excess NO can lead to oxidative stress and tissue damage, thereby contributing to chronic conditions, such as neurodegeneration or autoimmune disease (Nathan, 1992; Calabrese et al., 2007). In short, although constitutive NO production supports health, inducible NO can become a “double-edged sword” if regulation fails.

Synchronization of neural networks within the central nervous system requires energy metabolism, and thus effective and efficient mitochondrial function. Theta and gamma rhythms facilitate efficient communication and support critical cognitive processes. These oscillatory activities are energetically demanding; substantial quantities of ATP are required to maintain synaptic transmission, ion gradients, and neurotransmitter recycling. Mitochondria meet these demands through oxidative phosphorylation, thereby linking energy production to neural signaling. Metabolic regulatory factors, including the NAD⁺/NADH ratio and adenosine monophosphate-activated protein kinase, also modulate neuronal excitability and network synchrony, integrating cellular energy status with electrophysiological dynamics. Disruptions in mitochondrial function impair neural synchronization and contribute to cognitive decline in neurodegenerative diseases, while improved mitochondrial efficiency enhances network coherence. This reciprocal relationship suggests that energy metabolism is not only a substrate for brain activity but also a critical modulator of the brain’s functional connectivity (Attwell & Laughlin, 2001; Buzsáki & Draguhn, 2004; Fries, 2005; Kann et al., 2014; Yellen, 2018).

Notably, our previous studies highlighted the contributions of novel morphine-selective Mu-3 type opiate receptors on the inner mitochondrial membranes of human cells whose activities are coupled to NO release (Stefano, 1999; Stefano & Kream, 2009; Stefano et al., 2015); several reports describe the functional links between these receptors and the constitutive form of NOS, leading to responses previously attributed to NO alone (Esch et al., 2020; Toda et al., 2008; Toda et al., 2009; Mastronicola et al., 2004; Hervera et al., 2011). Other studies highlighted the selective impairment of neuronal Mu-type opiate receptors in response to mitochondrial oxidative damage (Raut et al., 2006; 2007).

The benefits of the relaxation response may indeed stem directly from its impact on mitochondria, which are organelles that are central to energy production. As discussed in the previous section, NO is a messenger/neurotransmitter and also a key regulatory molecule that plays an important role in modulating mitochondrial respiration and oxygen utilization and thus may play a critical role in promoting the relaxation response (Dusek et al., 2006; Stefano et al., 2006; Mantione et al., 2007). A large body of clinical and pre-clinical literature has established a key regulatory role for NO in maintaining normative rates of mitochondrial respiration and oxygen utilization (Stefano et al., 2019). Behaviorally mediated practices like the relaxation response and related mind-body interventions likely function effectively, because they support the synchronization of peripheral and central nervous systems based on their impact on mitochondrial function, leading to optimized ATP production. For instance, controlled breathing exercises associated with a relaxation response may enhance cortical-limbic integration, brainstem respiratory rhythms, and pulmonary gas exchange, further improving mitochondrial efficiency (Garner et al., 2019; Gothe et al., 2019). The physiological and clinical benefits of relaxation, particularly those facilitated by breathing-based mind-body practices, most likely emerge from a convergence of biochemical, neurological, and psychosocial pathways, with mitochondrial ATP optimization serving as a central mechanism. Slow, diaphragmatic breathing increases parasympathetic (vagal) tone and enhances oxygen availability, which in turn supports more efficient oxidative phosphorylation within the mitochondria. This leads to improved ATP synthesis, reduced production of reactive oxygen species, and stabilization of cellular redox states, all factors that are essential for maintaining energy homeostasis in neurons, immune cells, and endocrine tissues (Gautam et al., 2021a; 2021b).

**Fig. 2:**
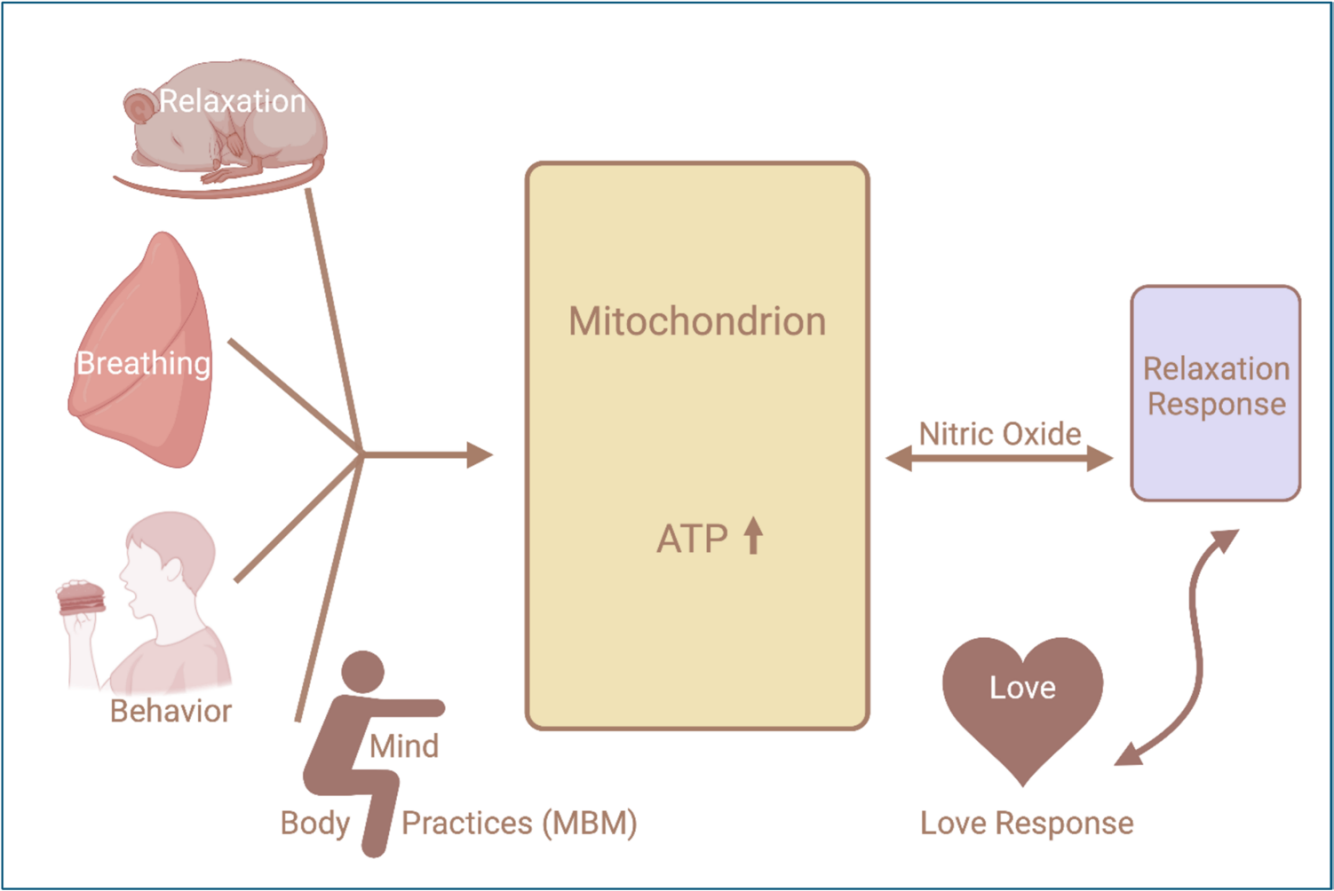
Interrelatedness of MBM and the relaxation/love responses via mitochondrial energy regulation. Explanations see text; MBM = mind/body medicine; ATP = adenosine triphosphate; NO = nitric oxide

Within our proposed working model, behaviorally mediated improvements of whole-body cellular bioenergetics result from the convergence of biochemical and biophysical processes within the mitochondrial matrix that lead to the optimized synthesis of ATP from ADP and inorganic phosphate via characterized chemiosmotic-driven events (Fig. 2). Furthermore, dynamic recycling of NO and inorganic nitrite by intramitochondrial nitrite reductases has been empirically demonstrated to represent a biochemical switching mechanism that is precisely regulated by minute variations in oxygen tension (Kream & Stefano, 2009; Stefano et al., 2019; Stefano & Kream, 2011; 2016).

## Love, stress, and reward

The deep significance of romantic bonding is reflected in the physiological response triggered by falling in love, which closely resembles a stress response, mobilizing physical energy and strength. Falling in love initiates a neurochemical cascade, including the release of stress hormones, such as rising cortisol levels (Esch & Stefano, 2005a; 2005b; Acevedo et al., 2012). Dopaminergic reward pathways become highly active and exhibit elevated testosterone levels in males. Studies have also found a decline in serotonin levels, which is similar to patterns observed in obsessive-compulsive disorder and depression (Marazziti et al., 2021). Over time, these neurochemical fluctuations stabilize: cortisol and serotonin levels return to baseline, testosterone decreases, while dopaminergic activity remains heightened. At this stage, the bonding neuropeptides oxytocin and vasopressin increase, reinforcing attachment and long-term connection (Esch & Stefano, 2005a; 2005b; Marazziti et al., 2021). The normalization of cortisol and adrenaline levels naturally decline as one feels secure in a relationship (Foerster & Kanske, 2022; Olds & Schwartz, 2023). Thus, love itself can be a source of stress (Stefano & Esch, 2005; Esch et al., 2024). Emotional pain, such as the loss of a loved one, activates the same brain regions involved in processing physical pain, demonstrating sharing (Kross et al., 2011). Importantly, individuals who describe themselves as “madly in love” with long-term partners continue to exhibit high activity in dopaminergic reward pathways, similar to the early stages of romantic love (Acevedo et al., 2012). At the same time, their brains show increased calming activity in regions rich in opioid and oxytocin receptors, suggesting a shift toward deeper emotional security and attachment (Acevedo et al., 2012; 2020). Furthermore, strong social connections contribute significantly to both mental and physical health (Esch et al., 2024).

Interestingly, the loss of love can have physiological consequences as well, such as broken heart syndrome (Wittstein et al., 2005). Many patients in Wittstein’s original study developed symptoms following the loss of a loved one, exhibiting signs resembling a heart attack or heart failure, including altered EKG patterns and elevated cardiac injury markers. However, unlike a typical heart attack caused by coronary artery disease, their arteries remained unobstructed. Instead, the underlying mechanism appears to involve an acute stress response, marked by excessive sympathetic nervous system activation and a surge of stress hormones that only temporarily weakens the heart muscle. Thus, broken heart syndrome demonstrates the intricate link between love and stress, highlighting the profound interplay between the brain’s reward and stress systems—again demonstrating the sharing of specific CNS pathways.

In a 2021 study, Tawakol and colleagues analyzed a large database of PET and CT scans from patients with and without broken heart syndrome (Radfar et al., 2021). The researchers examined brain images taken before individuals developed the condition in the amygdala, which represents an important brain region in the stress response (Salamon et al., 2005). Their findings revealed that broken heart syndrome exhibitors had heightened amygdala activity, indicating that the brain plays a role in the disorder (Radfar et al., 2021). Furthermore, those with the highest amygdala activity not only had a greater likelihood of developing the condition but also experienced its onset sooner than those with lower activity levels. Taken together, these findings underscore the deep interconnection between love, stress, neural pathways, and physiological health. Love and strong relationships do more than shape emotion; they actively regulate stress and promote well-being (Stefano et al., 2019; Esch et al., 2024). Therefore, mind and body are inextricably linked, reinforcing the impact of love on overall health. Its importance can hardly be overestimated and will certainly become even clearer in future research.

Relaxation strategies may also lead to improved mitochondrial function by regulating glucose metabolism and insulin secretion. As pancreatic β-cells are highly dependent on mitochondrial ATP generation to trigger insulin release in response to glucose, enhanced ATP production contributes directly to this signaling cascade (Maechler & Wollheim, 2001). At the same time, optimized mitochondrial function can limit inflammasome activation and suppress the production of proinflammatory cytokines by maintaining mitochondrial integrity and reducing oxidative stress. Finally, relaxation strategies also stimulate the vagus nerve, which independently limits inflammation via the cholinergic anti-inflammatory reflex, which is a mechanism that suppresses NF-κB activity and inflammatory cytokine expression that does not rely directly on ATP production (Tracey, 2002).

The psychological and emotional effects of relaxation also contribute to its physiological impact. Cognitive reappraisal, a core feature of many mind-body interventions, alters the impact of stressors and limits the activation of the hypothalamic-pituitary-adrenal axis. Downregulation of these responses results in lower cortisol levels and decreased sympathetic nervous system activity, which further protects mitochondrial function and improves systemic metabolic efficiency (Black & Slavich, 2016). Additionally, the practice of relaxation and mindfulness has been shown to increase dopamine availability in key brain regions associated with motivation, reward, and cognitive control. As a whole, these effects may indirectly enhance mitochondrial activity and reinforce adaptive neuroplasticity (Esch, 2014; Tang et al., 2015; Fu et al., 2017).

There are also several published studies that address the impact of relaxation techniques on glucose metabolism. Septimar and colleagues (2021) reported that diabetic patients who participated in progressive muscle exercises (i.e., Benson’s relaxation techniques) exhibited improved blood sugar levels compared with controls. Similarly, Gowri et al. (2022) reported significant improvements in glycemic control and diminished insulin resistance among diabetic patients participating in an integrated yoga therapy program.

Social and emotional cues, such as perceived safety and social support, also shape the physiological benefits of relaxation. Oxytocin and serotonin, neurochemicals associated with trust, bonding, and affective regulation, influence mitochondrial resilience and stress reactivity (Lee et al., 2018). These findings suggest that ATP optimization may be only one part of a broader integrative framework in which bioenergetics, neuroendocrine responses, immune signaling, and emotional-cognitive processing interact dynamically with one another.

Taken together, the benefits of relaxation cannot be attributed to a single mechanism. Rather, they emerge from the coordinated interaction of mitochondrial energy regulation, cognitive-emotional modulation, and autonomic recalibration. Adenosine Triphosphate optimization plays a foundational role in this network, as this mechanism supports the high metabolic demands associated with stress resilience and recovery, but its effects are amplified by the top-down influence of neural circuits governing perception, emotion, and social behavior. Thus, these interdependent mechanisms can help guide the design of integrative therapies that address both the cellular and psychological dimensions of chronic stress and metabolic imbalance.

Nitric oxide modulation has also been implicated as a central mediator in the positive outcomes of loving-kindness meditation (Kemper et al., 2014). In a previous study, we suggested that NO autoregulatory pathways coupled with endorphin and endogenous morphinergic mechanisms may play a central role in generating emotional “in love” responses (Esch & Stefano, 2005a; 2005b) (Fig. 2). Moreover, previous studies by Leza and colleagues (1996) revealed elevated levels of NOS in brain tissue during drug withdrawal and that administration of NOS inhibitors could attenuate the physical symptoms associated with opioid withdrawal (Adams et al., 1993; Majeed et al., 1994; Kimes et al., 1993). More recently, Kalamarides and colleagues (2024) found that the administration of a NOS inhibitor also limited the negative symptoms that frequently emerge after opioid withdrawal. These findings not only underscore a potentially important link between cellular bioenergetics and drug dependence, but they also suggest NO modulation as a potentially important target for drug development.

The seemingly contradictory roles of NOS can be clarified by recognizing the dose- and context-dependent effects of NO on mitochondrial function and neural health. Physiological levels of NO produced by constitutive forms of NOS (e.g., nNOS) can regulate mitochondrial respiration. In this setting, NO reversibly inhibits cytochrome c oxidase and helps to fine-tune ATP production, redox signaling, and neural plasticity (Brown & Cooper, 1994; Dromparis & Michelakis, 2012). However, during drug withdrawal or severe emotional stress, excessive NO production, often driven by upregulation of inducible NOS (iNOS), can lead to nitrosative stress, mitochondrial dysfunction, and neuronal damage secondary to elevated levels of peroxynitrite and other reactive nitrogen species (Itzhak et al., 2000; Cunha-Oliveira et al., 2008). Thus, therapeutic NOS modulation is not aimed at eliminating NO signaling entirely; instead, the goal is to restore NO levels to the physiological range to preserve mitochondrial function while preventing toxic overproduction. In this context, we hypothesize that relaxation-based therapies may help reestablish autonomic balance and normalize NOS activity, thereby supporting ATP optimization without the detrimental effects associated with NOS overactivation.

## Future directions

Among the directions for future research, we recognize that our current understanding of the links between cellular bioenergetics and the central reward pathway remains limited. Another topic of interest in this context is the role of gene transcription (Esch & Stefano, 2004). This phenomenon is of particular interest to those studying the impact of chronic drug use and addiction. Among recent findings, drug use leads to the upregulation of numerous transcription factors, including ΔFosB, cAMP response element binding protein, and nuclear factor-kappa B (NF-κB) (Crews et al., 2011; Nestler, 2012); some of these responses have been directly linked to NO activation (Xu et al., 2013; Winick-Ng et al., 2012; Hemish et al., 2003). Other genes implicated in this process include glucocorticoid receptor, NAc1 transcription factor, early growth response factors, and signal transducers and activators of transcription (Nestler, 2001; Nestler et al., 2012). Epigenetic changes that develop in response to long-term drug use have also been identified (Robison & Nestler, 2011). Equally intriguing are the findings of Murray and colleagues (2018), who reported differential expression of a wide variety of immune system genes, notably up-regulation of innate immune responses (e.g., type I interferons) in circulating leukocytes in a 2-year longitudinal study of young women engaging in new relationships.

## Conclusions

While many have recognized qualitative behavioral similarities between falling in love and drug addiction (i.e., falling in love with a fellow human being vs. falling “in love” with opiates), advances in functional imaging methods have permitted researchers to confirm many of the neurobiological links between these phenomena. Recent research has highlighted the brain’s reward system and its central role in events associated with both drug addiction and these interpersonal responses. Among these findings, we highlight the central role(s) played by NO in both signal transmission and bioenergetic modulation. Future studies might focus on further exploration of this critical dual role and likewise examine its links to gene transcription and epigenetic modulation in both neural and peripheral tissues.

## References

[CR1] Acevedo, B. P., Aron, A., Fisher, H. E., & Brown, L. L. (2012). Neural correlates of long-term intense romantic love. *Social Cognitive and Affective Neuroscience,**7*(2), 145–159. 10.1093/scan/nsq092 21208991 10.1093/scan/nsq092 PMC3277362

[CR2] Acevedo, B. P., Poulin, M. J., Collins, N. L., & Brown, L. L. (2020). After the honeymoon: Neural and genetic correlates of romantic love in newlywed marriages. *Frontiers in Psychology,**11*, 634. 10.3389/fpsyg.2020.00634 32457675 10.3389/fpsyg.2020.00634 PMC7223160

[CR3] Adams, M. L., Kalicki, J. M., Meyer, E. R., & Cicero, T. J. (1993). Inhibition of the morphine withdrawal syndrome by a nitric oxide synthase inhibitor, NG-nitro-L-arginine methyl ester. *Life Sciences,**52*(24), PL245–PL249. 10.1016/0024-3205(93)90472-f 7684108 10.1016/0024-3205(93)90472-f

[CR4] American Psychological Association. (2018). Romantic love. In *APA Dictionary of Psychology*. https://dictionary.apa.org/romantic-love

[CR5] American Psychological Association. (2018). Addiction. In *APA Dictionary of Psychology*. https://dictionary.apa.org/addiction

[CR6] American Society of Addiction Medicine. (2019). Definition of addiction. https://www.asam.org/quality-care/definition-of-addiction

[CR7] Aron, A., Fisher, H., Mashek, D. J., Strong, G., Li, H., & Brown, L. L. (2005). Reward, motivation, and emotion systems associated with early-stage intense romantic love. *Journal of Neurophysiology,**94*(1), 327–337. 10.1152/jn.00838.2004 15928068 10.1152/jn.00838.2004

[CR8] Attwell, D., & Laughlin, S. B. (2001). An energy budget for signaling in the grey matter of the brain. *Journal of Cerebral Blood Flow & Metabolism,**21*(10), 1133–1145. 10.1097/00004647-200110000-00001 11598490 10.1097/00004647-200110000-00001

[CR9] Beary, J. F., Benson, H., & Klemchuk, H. P. (1974). A simple psychophysiologic technique which elicits the hypometabolic changes of the relaxation response. *Psychosomatic Medicine,**36*(2), 115–120. 10.1097/00006842-197403000-00003 4814665 10.1097/00006842-197403000-00003

[CR10] Benson, H., Greenwood, M. M., & Klemchuk, H. (1975). The relaxation response: Psychophysiologic aspects and clinical applications. *The International Journal of Psychiatry in Medicine,**6*(1–2), 87–98. 10.2190/376W-E4MT-QM6Q-H0UM 773864 10.2190/376W-E4MT-QM6Q-H0UM

[CR11] Bhasin, M. K., Denninger, J. W., Huffman, J. C., Joseph, M. G., Niles, H., Chad-Friedman, E., Goldman, R., Buczynski-Kelley, B., Mahoney, B. A., Fricchione, G. L., Dusek, J. A., Benson, H., Zusman, R. M., & Libermann, T. A. (2018). Specific transcriptome changes associated with blood pressure reduction in hypertensive patients after relaxation response training. *The Journal of Alternative and Complementary Medicine,**24*(5), 486–504. 10.1089/acm.2017.0053 29616846 10.1089/acm.2017.0053 PMC5961875

[CR12] Bhasin, M. K., Dusek, J. A., Chang, B.-H., Joseph, M. G., Denninger, J. W., Fricchione, G. L., Benson, H., & Libermann, T. A. (2013). Relaxation response induces temporal transcriptome changes in energy metabolism, insulin secretion and inflammatory pathways. *PLoS One,**8*(5), Article e62817. 10.1371/journal.pone.0062817 23650531 10.1371/journal.pone.0062817 PMC3641112

[CR13] Bressan, P., & Kramer, P. (2021). Mental health, mitochondria, and the battle of the sexes. *Biomedicines,**9*(2), 116. 10.3390/biomedicines9020116 33530498 10.3390/biomedicines9020116 PMC7911591

[CR14] Black, D. S., & Slavich, G. M. (2016). Mindfulness meditation and the immune system: A systematic review of randomized controlled trials. *Annals of the New York Academy of Sciences,**1373*(1), 13–24. 10.1111/nyas.12998 26799456 10.1111/nyas.12998 PMC4940234

[CR15] Brown, G. C., & Cooper, C. (1994). Nanomolar concentrations of nitric oxide reversibly inhibit synaptosomal respiration by competing with oxygen at cytochrome oxidase. *FEBS Letters,**356*(2–3), 295–298. 10.1016/0014-5793(94)01290-3 7805858 10.1016/0014-5793(94)01290-3

[CR16] Brown, R. P., & Gerbarg, P. L. (2005). Sudarshan kriya yogic breathing in the treatment of stress, anxiety, and depression: Part II—clinical applications and guidelines. *The Journal of Alternative and Complementary Medicine,**11*(4), 711–717. 10.1089/acm.2005.11.711 16131297 10.1089/acm.2005.11.711

[CR17] Burkett, J. P., Spiegel, L. L., Inoue, K., Murphy, A. Z., & Young, L. J. (2011). Activation of μ-opioid receptors in the dorsal striatum is necessary for adult social attachment in monogamous prairie voles. *Neuropsychopharmacology,**36*(11), 2200–2210. 10.1038/npp.2011.117 21734650 10.1038/npp.2011.117 PMC3176565

[CR18] Buzsáki, G., & Draguhn, A. (2004). Neuronal oscillations in cortical networks. *Science,**304*(5679), 1926–1929. 10.1126/science.1099745 15218136 10.1126/science.1099745

[CR19] Calabrese, V., Mancuso, C., Calvani, M., Rizzarelli, E., Butterfield, D. A., & Stella, A. M. G. (2007). Nitric oxide in the central nervous system: Neuroprotection versus neurotoxicity. *Nature Reviews. Neuroscience,**8*(10), 766–775. 10.1038/nrn2214 17882254 10.1038/nrn2214

[CR20] Carlsson, A. (2000). *A half-century of neurotransmitter research: Impact on neurology and psychiatry* [Nobel lecture]. NobelPrize.org. https://www.nobelprize.org/prizes/medicine/2000/carlsson/lecture/ (Last accessed January 6, 2025)

[CR21] Carter, C. S. (2017). The oxytocin–vasopressin pathway in the context of love and fear. *Frontiers in Endocrinology, 8*, Article 356. 10.3389/fendo.2017.00356 10.3389/fendo.2017.00356 PMC5743651 29312146

[CR22] Crews, F. T., Zou, J., & Qin, L. (2011). Induction of innate immune genes in brain create the neurobiology of addiction. *Brain, Behavior, and Immunity,**25*(Suppl. 1), S4–S12. 10.1016/j.bbi.2011.03.003 21402143 10.1016/j.bbi.2011.03.003 PMC3552373

[CR23] Cunha-Oliveira, T., Rego, A. C., & Oliveira, C. R. (2008). Cellular and molecular mechanisms involved in the neurotoxicity of opioid and psychostimulant drugs. *Brain Research Reviews,**58*(1), 192–208. 10.1016/j.brainresrev.2008.03.002 18440072 10.1016/j.brainresrev.2008.03.002

[CR24] Dhaliwal, A., & Gupta, M. (2023). Physiology, opioid receptor. In *StatPearls*. StatPearls Publishing. https://www.ncbi.nlm.nih.gov/books/NBK546642/ (last accessed January 6, 2025) 31536249

[CR25] Dromparis, P., & Michelakis, E. D. (2012). Mitochondria in vascular health and disease. *Annual Review of Physiology,**75*(1), 95–126. 10.1146/annurev-physiol-030212-183804 23157555 10.1146/annurev-physiol-030212-183804

[CR26] Dusek, J. A., Chang, B. H., Zaki, J., Lazar, S., Deykin, A., Stefano, G. B., Wohlhueter, A. L., Hibberd, P. L., & Benson, H. (2006). Association between oxygen consumption and nitric oxide production during the relaxation response. *Medical Science Monitor,**12*, CR1–CR10. 16369463

[CR27] Epstein, F. H., Moncada, S., & Higgs, A. (1993). The L-arginine-nitric oxide pathway. *New England Journal of Medicine,**329*(27), 2002–2012. 10.1056/nejm199312303292706 7504210 10.1056/NEJM199312303292706

[CR28] Esch, T., & Stefano, G. B. (2004). The neurobiology of pleasure, reward processes, addiction and their health implications. *Neuroendocrinology Letters,**25*(4), 235–251. 15361811

[CR29] Esch, T., & Stefano, G. B. (2005). The neurobiology of love. *Neuroendocrinology Letters,**26*(3), 175–192. 15990719

[CR30] Esch, T., & Stefano, G. B. (2005). Love promotes health. *Neuroendocrinology Letters,**26*(3), 264–267. 15990734

[CR31] Esch, T., & Stefano, G. B. (2010). Endogenous reward mechanisms and their importance in stress reduction, exercise and the brain. *Archives of Medical Science,**6*(3), 447–455. 10.5114/aoms.2010.14269 22371784 10.5114/aoms.2010.14269 PMC3282525

[CR32] Esch, T. (2014). The neurobiology of meditation and mindfulness. In S. Schmidt spsampsps H. Walach (Eds.), *Meditation – Neuroscientific approaches and philosophical implications* (Vol. 2, pp. 153–173). Springer. 10.1007/978-3-319-01634-4_9

[CR33] Esch, T., Kream, R. M., & Stefano, G. B. (2020). Emerging regulatory roles of opioid peptides, endogenous morphine, and opioid receptor subtypes in immunomodulatory processes: Metabolic, behavioral, and evolutionary perspectives. *Immunology Letters,**227*, 28–33. 10.1016/j.imlet.2020.08.007 32827633 10.1016/j.imlet.2020.08.007

[CR34] Esch, T., Stefano, G. B., & Michaelsen, M. M. (2024). The foundations of mind-body medicine: Love, good relationships, and happiness modulate stress and promote health. *Stress and Health,**40*(1), Article e3387. 10.1002/smi.3387 38442034 10.1002/smi.3387

[CR35] Fisher, H., Aron, A., & Brown, L. L. (2005). Romantic love: An fMRI study of a neural mechanism for mate choice. *The Journal of Comparative Neurology,**493*(1), 58–62. 10.1002/cne.20772 16255001 10.1002/cne.20772

[CR36] Fisher, H. E., Xu, X., Aron, A., & Brown, L. L. (2016). Intense, passionate, romantic love: A natural addiction? How the fields that investigate romance and substance abuse can inform each other. *Frontiers in Psychology,**7*, 687. 10.3389/fpsyg.2016.00687 27242601 10.3389/fpsyg.2016.00687 PMC4861725

[CR37] Foerster, K., & Kanske, P. (2022). Upregulating positive affect through compassion: Psychological and physiological evidence. *International Journal of Psychophysiology,**176*, 100–107. 10.1016/j.ijpsycho.2022.03.009 35358613 10.1016/j.ijpsycho.2022.03.009

[CR38] Fries, P. (2005). A mechanism for cognitive dynamics: Neuronal communication through neuronal coherence. *Trends in Cognitive Sciences,**9*(10), 474–480. 10.1016/j.tics.2005.08.011 16150631 10.1016/j.tics.2005.08.011

[CR39] Fu, Z. X., Tan, X., Fang, H., Lau, P. M., Wang, X., Cheng, H., & Bi, G. Q. (2017). Dendritic mitoflash as a putative signal for stabilizing long-term synaptic plasticity. *Nature Communications,**8*(1), 31. 10.1038/s41467-017-00043-3 28652625 10.1038/s41467-017-00043-3 PMC5484698

[CR40] Furlan, A., & Petrus, P. (2023). Brain–body communication in metabolic control. *Trends in Endocrinology and Metabolism,**34*(12), 813–822. 10.1016/j.tem.2023.08.014 37716877 10.1016/j.tem.2023.08.014

[CR41] Garner, M., Reith, W., & Krick, C. (2019). 10-week Hatha yoga increases right hippocampal density compared to active and passive control groups: A controlled structural cMRI study. *Journal of Neuroimaging in Psychiatry and Neurology,* Article Article 027. 10.17756/jnpn.2019-027

[CR42] Gautam, S., Saxena, R., Dada, T., & Dada, R. (2021). Yoga—impact on mitochondrial health: Clinical consequences. *Annals of Neurosciences,**28*(3–4), 114–116. 10.1177/09727531211009431 35341229 10.1177/09727531211009431 PMC8948330

[CR43] Gautam, S., Kumar, U., Kumar, M., Rana, D., & Dada, R. (2021). Yoga improves mitochondrial health and reduces severity of autoimmune inflammatory arthritis: A randomized controlled trial. *Mitochondrion,**58*, 147–159. 10.1016/j.mito.2021.03.004 33741520 10.1016/j.mito.2021.03.004

[CR44] Gothe, N. P., Khan, I., Hayes, J., et al. (2019). Yoga effects on brain health: A systematic review of the current literature. *Brain Plasticity,**5*(1), 105–122. 10.3233/bpl-190084 31970064 10.3233/BPL-190084 PMC6971819

[CR45] Gowri, M. M., Rajendran, J., Srinivasan, A., Bhavanani, A. B., & Meena, R. (2022). Impact of an integrated yoga therapy protocol on insulin resistance and glycemic control in patients with type 2 diabetes mellitus. *Rambam Maimonides Medical Journal,**13*(1), Article e0005. 10.5041/rmmj.10462 35089124 10.5041/RMMJ.10462 PMC8798588

[CR46] Hemish, J., Nakaya, N., Mittal, V., & Enikolopov, G. (2003). Nitric oxide activates diverse signaling pathways to regulate gene expression. *Journal of Biological Chemistry,**278*(42), 42321–42329. 10.1074/jbc.m308192200 12907672 10.1074/jbc.M308192200

[CR47] Hervera, A., Negrete, R., Leánez, S., et al. (2011). Peripheral effects of morphine and expression of μ-opioid receptors in the dorsal root ganglia during neuropathic pain: Nitric oxide signaling. *Molecular Pain,**7*, 25. 10.1186/1744-8069-7-25 21486477 10.1186/1744-8069-7-25 PMC3094254

[CR48] Insel, T. R. (2010). The challenge of translation in social neuroscience: A review of oxytocin, vasopressin, and affiliative behavior. *Neuron,**65*(6), 768–779. 10.1016/j.neuron.2010.03.005 20346754 10.1016/j.neuron.2010.03.005 PMC2847497

[CR49] Itzhak, Y., Martin, J. L., & Ali, S. F. (2000). Comparison between the role of the neuronal and inducible nitric oxide synthase in methamphetamine-induced neurotoxicity and sensitization. *Annals of the New York Academy of Sciences,**914*(1), 104–111. 10.1111/j.1749-6632.2000.tb05188.x 11085313 10.1111/j.1749-6632.2000.tb05188.x

[CR50] Iversen, S. D., & Iversen, L. L. (2007). Dopamine: 50 years in perspective. *Trends in Neurosciences,**30*, 188–193. 10.1016/j.tins.2007.03.002 17368565 10.1016/j.tins.2007.03.002

[CR51] Kalamarides, D. J., Singh, A., & Dani, J. A. (2024). Protracted opioid withdrawal behaviors are reduced by nitric oxide inhibition in mice. *Addiction Neuroscience,**12*, Article 100167. 10.1016/j.addicn.2024.100167

[CR52] Kann, O., Papageorgiou, I. E., & Draguhn, A. (2014). Highly energized inhibitory interneurons are a central element for information processing in cortical networks. *Journal of Cerebral Blood Flow & Metabolism,**34*(8), 1270–1282. 10.1038/jcbfm.2014.104 24896567 10.1038/jcbfm.2014.104 PMC4126088

[CR53] Karrasch, S., Mavioğlu, R. N., Matits, L., et al. (2023). Randomized controlled trial investigating potential effects of relaxation on mitochondrial function in immune cells: A pilot experiment. *Biological Psychology,**183*, Article 108656. 10.1016/j.biopsycho.2023.108656 37544424 10.1016/j.biopsycho.2023.108656

[CR54] Kemper, K. J., Powell, D., Helms, C. C., & Kim-Shapiro, D. B. (2014). Loving-kindness meditation’s effects on nitric oxide and perceived well-being: A pilot study in experienced and inexperienced meditators. *Explore,**11*(1), 32–39. 10.1016/j.explore.2014.10.002 25457445 10.1016/j.explore.2014.10.002

[CR55] Kimes, A. S., Vaupel, D. B., & London, E. D. (1993). Attenuation of some signs of opioid withdrawal by inhibitors of nitric oxide synthase. *Psychopharmacology,**112*(4), 521–524. 10.1007/bf02244904 7532866 10.1007/BF02244904

[CR56] Klajner, F., Hartman, L. M., & Sobell, M. B. (1984). Treatment of substance abuse by relaxation training: A review of its rationale, efficacy and mechanisms. *Addictive Behaviors,**9*(1), 41–55. 10.1016/0306-4603(84)90006-6 6377844 10.1016/0306-4603(84)90006-6

[CR57] Koob, G. F., & Volkow, N. D. (2016). Neurobiology of addiction: A neurocircuitry analysis. *The Lancet Psychiatry,**3*(8), 760–773. 10.1016/s2215-0366(16)00104-8 27475769 10.1016/S2215-0366(16)00104-8 PMC6135092

[CR58] Kream, R. M., & Stefano, G. B. (2009). Endogenous morphine and nitric oxide coupled regulation of mitochondrial processes. *Medical Science Monitor,**15*(5), RA263–RA268. 19946245

[CR59] Kross, E., Berman, M. G., Mischel, W., et al. (2011). Social rejection shares somatosensory representations with physical pain. *Proceedings of the National Academy of Sciences of the United States of America,**108*(15), 6270–6275. 10.1073/pnas.1102693108 21444827 10.1073/pnas.1102693108 PMC3076808

[CR60] Lee, M. R., Scheidweiler, K. B., Diao, X. X., Akhlaghi, F., Cummins, A., Huestis, M. A., Leggio, L., & Averbeck, B. B. (2018). Oxytocin by intranasal and intravenous routes reaches the cerebrospinal fluid in rhesus macaques: Determination using a novel oxytocin assay. *Molecular Psychiatry,**23*(1), 115–122. 10.1038/mp.2017.27 28289281 10.1038/mp.2017.27 PMC5862033

[CR61] Le Merrer, J., Becker, J. A. J., Befort, K., & Kieffer, B. L. (2009). Reward processing by the opioid system in the brain. *Physiological Reviews,**89*(4), 1379–1412. 10.1152/physrev.00005.2009 19789384 10.1152/physrev.00005.2009 PMC4482114

[CR62] Lewis, R. G., Florio, E., Punzo, D., spsampsps Borrelli, E. (2021). The brain’s reward system in health and disease. In *Advances in Experimental Medicine and Biology* (Vol. 1344, pp. 57–69). Springer. 10.1007/978-3-030-81147-1_4 10.1007/978-3-030-81147-1_4 PMC8992377 34773226

[CR63] Leza, J. C., Lizasoain, I., Cuellar, B., et al. (1996). Correlation between brain nitric oxide synthase activity and opiate withdrawal. *Naunyn-Schmiedeberg’s Archives of Pharmacology,**353*(3), 349–354. 10.1007/bf00168639 8692292 10.1007/BF00168639

[CR64] Lotfinia, S., Yaseri, A., Jamshidmofid, P., et al. (2024). Effect of relaxation-based virtual reality on psychological and physiological stress of substance abusers under detoxification: A randomized controlled trial. *Brain and Behavior,**14*, Article e70084. 10.1002/brb3.70084 39402817 10.1002/brb3.70084 PMC11473653

[CR65] Maechler, P., & Wollheim, C. B. (2001). Mitochondrial function in normal and diabetic β-cells. *Nature,**414*(6865), 807–812. 10.1038/414807a 11742413 10.1038/414807a

[CR66] Majeed, N. H., Przewłocka, B., Machelska, H., & Przewłocki, R. (1994). Inhibition of nitric oxide synthase attenuates the development of morphine tolerance and dependence in mice. *Neuropharmacology,**33*(2), 189–192. 10.1016/0028-3908(94)90006-x 7518573 10.1016/0028-3908(94)90006-x

[CR67] Mantione, K. J., Esch, T., & Stefano, G. B. (2007). Detection of nitric oxide in exhaled human breath: Exercise and resting determinations. *Medical Science Monitor,**13*(1), MT1–MT5. 17325642

[CR68] Marazziti, D., Palermo, S., spsampsps Mucci, F. (2021). The science of love: State of the art. In *Advances in Experimental Medicine and Biology* (Vol. 1331, pp. 249–254). Springer. 10.1007/978-3-030-74046-7_16 10.1007/978-3-030-74046-7_16 34453303

[CR69] Mastronicola, D., Arcuri, E., Arese, M., et al. (2004). Morphine but not fentanyl and methadone affects mitochondrial membrane potential by inducing nitric oxide release in glioma cells. *Cellular and Molecular Life Sciences,**61*, 2991–2997. 10.1007/s00018-004-4371-x 15583861 10.1007/s00018-004-4371-x PMC11924496

[CR70] Murnane, K. S., Edinoff, A. N., Cornett, E. M., & Kaye, A. D. (2023). Updated perspectives on the neurobiology of substance use disorders using neuroimaging. *Substance Abuse and Rehabilitation,**14*, 99–111. 10.2147/sar.s362861 37583934 10.2147/SAR.S362861 PMC10424678

[CR71] Murray, D. R., Haselton, M. G., Fales, M., & Cole, S. W. (2018). Falling in love is associated with immune system gene regulation. *Psychoneuroendocrinology,**100*, 120–126. 10.1016/j.psyneuen.2018.09.043 30299259 10.1016/j.psyneuen.2018.09.043 PMC6333523

[CR72] Nathan, C. (1992). Nitric oxide as a secretory product of mammalian cells. *FASEB Journal,**6*(12), 3051–3064. 1381691

[CR73] National Institute on Drug Abuse. (2007). *The neurobiology of drug addiction*. https://nida.nih.gov/sites/default/files/1922-the-neurobiology-of-drug-addiction.pdf

[CR74] Nestler, E. J. (2001). Molecular basis of long-term plasticity underlying addiction. *Nature Reviews Neuroscience,**2*(2), 119–128. 10.1038/35053570 11252991 10.1038/35053570

[CR75] Nestler, E. J. (2012). Transcriptional mechanisms of drug addiction. *Clinical Psychopharmacology and Neuroscience,**10*(3), 136–143. 10.9758/cpn.2012.10.3.136 23430970 10.9758/cpn.2012.10.3.136 PMC3569166

[CR76] Olds, J., & Schwartz, R. S. (2023). Why don’t you take this to a friend? A question psychotherapists should ask more often. *Harvard Review of Psychiatry,**31*(1), 47–49. 10.1097/HRP.0000000000000359 36884036 10.1097/HRP.0000000000000359 PMC9997617

[CR77] Peele, S., & Brodsky, A. (1975). *Love and addiction*. Taplinger Publishing.

[CR78] Peris, J., MacFadyen, K., Smith, J. A., et al. (2016). Oxytocin receptors are expressed on dopamine and glutamate neurons in the mouse ventral tegmental area that project to nucleus accumbens and other mesolimbic targets. *The Journal of Comparative Neurology,**525*(5), 1094–1108. 10.1002/cne.24116 27615433 10.1002/cne.24116 PMC6483090

[CR79] Picard, M., McEwen, B. S., Epel, E. S., & Sandi, C. (2018). An energetic view of stress: Focus on mitochondria. *Frontiers in Neuroendocrinology,**49*, 72–85. 10.1016/j.yfrne.2018.01.001 29339091 10.1016/j.yfrne.2018.01.001 PMC5964020

[CR80] Priest, C., & Tontonoz, P. (2019). Inter-organ cross-talk in metabolic syndrome. *Nature Metabolism,**1*(12), 1177–1188. 10.1038/s42255-019-0145-5J 32694672 10.1038/s42255-019-0145-5

[CR81] Radfar, A., Abohashem, S., Osborne, M. T., et al. (2021). Stress-associated neurobiological activity associates with the risk for and timing of subsequent Takotsubo syndrome. *European Heart Journal,**42*(19), 1898–1908. 10.1093/eurheartj/ehab029 33768230 10.1093/eurheartj/ehab029 PMC8121551

[CR82] Raut, A., Iglewski, M., & Ratka, A. (2006). Differential effects of impaired mitochondrial energy production on the function of mu and delta opioid receptors in neuronal SK-N-SH cells. *Neuroscience Letters,**404*(3), 242–246. 10.1016/j.neulet.2006.05.055 16808998 10.1016/j.neulet.2006.05.055

[CR83] Raut, A., Rao, V. R., & Ratka, A. (2007). Changes in opioid receptor proteins during mitochondrial impairment in differentiated SK-N-SH cells. *Neuroscience Letters,**422*(3), 187–192. 10.1016/j.neulet.2007.06.015 17611027 10.1016/j.neulet.2007.06.015 PMC2112745

[CR84] Rigney, N., De Vries, G. J., Petrulis, A., & Young, L. J. (2022). Oxytocin, vasopressin, and social behavior: From neural circuits to clinical opportunities. *Endocrinology,**163*(4), bqac111. 10.1210/endocr/bqac111 35863332 10.1210/endocr/bqac111 PMC9337272

[CR85] Rinne, P., Lahnakoski, J. M., Saarimäki, H., et al. (2024). Six types of loves differentially recruit reward and social cognition brain areas. *Cerebral Cortex,**34*, Article bhae331. 10.1093/cercor/bhae331 39183646 10.1093/cercor/bhae331 PMC11345515

[CR86] Robison, A. J., & Nestler, E. J. (2011). Transcriptional and epigenetic mechanisms of addiction. *Nature Reviews Neuroscience,**12*(11), 623–637. 10.1038/nrn3111 21989194 10.1038/nrn3111 PMC3272277

[CR87] Roth-Deri, I., Green-Sadan, T., & Yadid, G. (2008). β-Endorphin and drug-induced reward and reinforcement. *Progress in Neurobiology,**86*(1), 1–21. 10.1016/j.pneurobio.2008.06.003 18602444 10.1016/j.pneurobio.2008.06.003

[CR88] Rysztak, L. G., & Jutkiewicz, E. M. (2022). The role of enkephalinergic systems in substance use disorders. *Frontiers in Systems Neuroscience,**16*, Article 932546. 10.3389/fnsys.2022.932546 35993087 10.3389/fnsys.2022.932546 PMC9391026

[CR89] Salamon, E., Esch, T., & Stefano, G. B. (2005). Role of amygdala in mediating sexual and emotional behavior via coupled nitric oxide release. *Acta Pharmacologica Sinica,**26*(3), 389–395. 10.1111/j.1745-7254.2005.00083.x 15780186 10.1111/j.1745-7254.2005.00083.x

[CR90] Septimar, Z. M., Priatna, H., & Tomi, S. Y. (2021). Effect of Benson’s relaxation techniques on blood glucose levels in patients with diabetes mellitus. *Enfermería Clínica,**31*, S454–S456. 10.1016/j.enfcli.2020.09.044

[CR91] Seshadri, K. (2016). The neuroendocrinology of love. *Indian Journal of Endocrinology and Metabolism,**20*(4), 558. 10.4103/2230-8210.183479 27366726 10.4103/2230-8210.183479 PMC4911849

[CR92] Shyu, C., Chavez, S., Boileau, I., & Foll, B. L. (2022). Quantifying GABA in addiction: A review of proton magnetic resonance spectroscopy studies. *Brain Sciences,**12*(7), 918. 10.3390/brainsci12070918 35884725 10.3390/brainsci12070918 PMC9316447

[CR93] Song, H., Zou, Z., Kou, J., et al. (2015). Love-related changes in the brain: A resting-state functional magnetic resonance imaging study. *Frontiers in Human Neuroscience,**9*, 71. 10.3389/fnhum.2015.00071 25762915 10.3389/fnhum.2015.00071 PMC4327739

[CR94] Stefano, G. B. (1999). The Mu3 opiate receptor subtype. *Pain Forum,**8*(4), 206–209.

[CR95] Stefano, G. B., Fricchione, G. L., & Esch, T. (2006). Relaxation: Molecular and physiological significance. *Medical Science Monitor, 12*(4), HY21–HY31. 16940938

[CR96] Stefano, G. B., Esch, T., & Kream, R. M. (2019). Augmentation of whole-body metabolic status by mind-body training: Synchronous integration of tissue- and organ-specific mitochondrial function. *Medical Science Monitor Basic Research,**25*, 8–14. 10.12659/msmbr.913264 30631032 10.12659/MSMBR.913264 PMC6505060

[CR97] Stefano, G. B., & Esch, T. (2005). Love and stress. *Neuroendocrinology Letters,**26*(2), 173–174. 15990718

[CR98] Stefano, G. B., Goumon, Y., Bilfinger, T. V., Welters, I. D., & Cadet, P. (2000). Basal nitric oxide limits immune, nervous and cardiovascular excitation: Human endothelia express a mu opiate receptor. *Progress in Neurobiology,**60*(6), 513–530. 10.1016/s0301-0082(99)00038-6 10739087 10.1016/s0301-0082(99)00038-6

[CR99] Stefano, G. B., & Kream, R. M. (2009). Dopamine, morphine, and nitric oxide: An evolutionary signaling triad. *CNS Neuroscience & Therapeutics,**16*(6), e124–e137. 10.1111/j.1755-5949.2009.00114.x 19912274 10.1111/j.1755-5949.2009.00114.x PMC6493803

[CR100] Stefano, G. B., & Kream, R. M. (2011). Reciprocal regulation of cellular nitric oxide formation by nitric oxide synthase and nitrite reductases. *Medical Science Monitor,**17*(7), RA221–RA226. 10.12659/msm.881972 21959625 10.12659/MSM.881972 PMC3539480

[CR101] Stefano, G. B., & Kream, R. M. (2016). Dysregulated mitochondrial and chloroplast bioenergetics from a translational medical perspective. *International Journal of Molecular Medicine,**37*(3), 547–555. 10.3892/ijmm.2016.2471 26821064 10.3892/ijmm.2016.2471 PMC4771107

[CR102] Stefano, G. B., & Kream, R. M. (2017). Aging reversal and healthy longevity is in reach: Dependence on mitochondrial DNA heteroplasmy as a key molecular target. *Medical Science Monitor,**23*, 2732–2735. 10.12659/MSM.902515 28579605 10.12659/MSM.902515 PMC5470867

[CR103] Stefano, G. B., Mantione, K. J., Capellan, L., Casares, F. M., Challenger, S., Ramin, R., Samuel, J. M., Snyder, C., & Kream, R. M. (2015). Morphine stimulates nitric oxide release in human mitochondria. *Journal of Bioenergetics and Biomembranes,**47*(5), 409–417. 10.1007/s10863-015-9626-8 26350413 10.1007/s10863-015-9626-8

[CR104] Stefano, G. B., Murga, J., Benson, H., Zhu, W., Bilfinger, T. V., & Magazine, H. I. (2001). Nitric oxide inhibits norepinephrine stimulated contraction of human internal thoracic artery and rat aorta. *Pharmacological Research,**43*(2), 199–203. 10.1006/phrs.2000.0765 11243723 10.1006/phrs.2000.0765

[CR105] Stefano, G. B., Ptáček, R., Kuželová, H., & Kream, R. M. (2012). Endogenous morphine: Up-to-date review 2011. *Folia Biologica,**58*(2), 49–56. 22578954 10.14712/fb2012058020049

[CR106] Tang, Y., Hölzel, B. K., & Posner, M. I. (2015). The neuroscience of mindfulness meditation. *Nature Reviews. Neuroscience,**16*(4), 213–225. 10.1038/nrn3916 25783612 10.1038/nrn3916

[CR107] Toda, N., Kishioka, S., Hatano, Y., et al. (2008). Modulation of opioid actions by nitric oxide signaling. *Anesthesiology,**110*(1), 166–181. 10.1097/aln.0b013e31819146a9 10.1097/ALN.0b013e31819146a9 19104184

[CR108] Toda, N., Kishioka, S., Hatano, Y., & Toda, H. (2009). Interactions between morphine and nitric oxide in various organs. *Journal of Anesthesia,**23*(4), 554–568. 10.1007/s00540-009-0793-9 19921366 10.1007/s00540-009-0793-9

[CR109] Tomkins, D. M., & Sellers, E. M. (2001). Addiction and the brain: The role of neurotransmitters in the cause and treatment of drug dependence. *Canadian Medical Association Journal,**164*(6), 817–821. 11276551 PMC80880

[CR110] Tracey, K. J. (2002). The inflammatory reflex. *Nature,**420*(6917), 853–859. 10.1038/nature01321 12490958 10.1038/nature01321

[CR111] Valentino, R. J., & Volkow, N. D. (2018). Untangling the complexity of opioid receptor function. *Neuropsychopharmacology,**43*(13), 2514–2520. 10.1038/s41386-018-0225-3 30250308 10.1038/s41386-018-0225-3 PMC6224460

[CR112] Volkow, N. D., Fowler, J. S., & Wang, G.-J. (2003). The addicted human brain: Insights from imaging studies. *Journal of Clinical Investigation,**111*(10), 1444–1451. 10.1172/jci200318533 12750391 10.1172/JCI18533 PMC155054

[CR113] Volkow, N. D., Fowler, J. S., Wang, G.-J., et al. (2007). Dopamine in drug abuse and addiction. *Archives of Neurology,**64*(11), Article 1575. 10.1001/archneur.64.11.1575 17998440 10.1001/archneur.64.11.1575

[CR114] Volkow, N. D., Michaelides, M., & Baler, R. (2019). The neuroscience of drug reward and addiction. *Physiological Reviews,**99*(4), 2115–2140. 10.1152/physrev.00014.2018 31507244 10.1152/physrev.00014.2018 PMC6890985

[CR115] Winick-Ng, W., Leri, F., & Kalisch, B. E. (2012). Nitric oxide and histone deacetylases modulate cocaine-induced mu-opioid receptor levels in PC12 cells. *BMC Pharmacology and Toxicology,**13*(1), 11. 10.1186/2050-6511-13-11 23079001 10.1186/2050-6511-13-11 PMC3520874

[CR116] Wittstein, I. S., Thiemann, D. R., Lima, J. A., et al. (2005). Neurohumoral features of myocardial stunning due to sudden emotional stress. *New England Journal of Medicine,**352*(6), 539–548. 10.1056/NEJMoa043046 15703419 10.1056/NEJMoa043046

[CR117] Wronikowska-Denysiuk, O., Mrozek, W., & Budzyńska, B. (2023). The role of oxytocin and vasopressin in drug-induced reward—Implications for social and non-social factors. *Biomolecules,**13*(3), 405. 10.3390/biom13030405 36979340 10.3390/biom13030405 PMC10046619

[CR118] Xu, R., Serritella, A. V., Sen, T., et al. (2013). Behavioral effects of cocaine mediated by nitric oxide-GAPDH transcriptional signaling. *Neuron,**78*(4), 623–630. 10.1016/j.neuron.2013.03.021 23719162 10.1016/j.neuron.2013.03.021 PMC4047707

[CR119] Ye, J., & Medzhitov, R. (2019). Control strategies in systemic metabolism. *Nature Metabolism,**1*(10), 947–957. 10.1038/s42255-019-0118-8 32694839 10.1038/s42255-019-0118-8

[CR120] Yellen, G. (2018). Fueling thought: Management of glycolysis and oxidative phosphorylation in neuronal metabolism. *The Journal of Cell Biology,**217*(7), 2235–2246. 10.1083/jcb.201803152 29752396 10.1083/jcb.201803152 PMC6028533

[CR121] Zalewska-Kaszubska, J., & Czarnecka, E. (2005). Deficit in beta-endorphin peptide and tendency to alcohol abuse. *Peptides,**26*(4), 701–705. 10.1016/j.peptides.2004.11.010 15752586 10.1016/j.peptides.2004.11.010

[CR122] Zeki, S. (2007). The neurobiology of love. *FEBS Letters,**581*(14), 2575–2579. 10.1016/j.febslet.2007.03.094 17531984 10.1016/j.febslet.2007.03.094

[CR123] Zhu, W., Cadet, P., Baggerman, G., Mantione, K. J., & Stefano, G. B. (2005). Human white blood cells synthesize morphine: CYP2D6 modulation. *Journal of Immunology,**175*(11), 7357–7362. 10.4049/jimmunol.175.11.7357 10.4049/jimmunol.175.11.7357 16301642

